# Modeling thermophysical properties of glasses

**DOI:** 10.1038/s41598-023-27747-5

**Published:** 2023-01-18

**Authors:** Angelo Lucia, Otto Gregory

**Affiliations:** Department of Chemical Engineering, University of Rhode Island, 2 East Alumni Avenue, Kingston, RI 02881 USA

**Keywords:** Engineering, Materials science, Mathematics and computing

## Abstract

Metal oxide glasses are important in various industries because their properties can be tailored to meet application-specific requirements. However, there are few rigorous modeling tools for predicting thermomechanical properties of these materials with acceptable accuracy and speed, yet these properties can play a critical role in material design. In this article, a general multi-scale modeling framework based on Monte Carlo simulation and a cubic equation of state for predicting thermomechanical properties is presented. There are two novel and fundamental aspects of this work: (1) characterization of glass transition and softening temperatures as adjacent saddle points on the heat capacity versus temperature curve, and (2) a new moving boundary equation of state that accounts for structure and ‘soft’ repulsion. In addition, modeling capabilities are demonstrated by comparing thermomechanical properties of a pure B_2_O_3_ glass and PbO–B_2_O_3_ glass predicted by the equation of state to experimental data. Finally, this work provides a rigorous approach to estimating thermophysical properties for the purpose of guiding experimental work directed at tailoring thermomechanical properties of glasses to fit applications.

## Introduction

Metal oxide glasses (or glass–ceramics) are important industrial materials that exhibit a unique set of thermal, mechanical, and optical properties. They can also provide excellent chemical resistance and are used in a wide range of applications that include adding color, iridescence, and luster to glass, in semi-conductor materials processing, for thermal imaging as thermal paints, optical communication, chemical sensors, hermetic glass-metal seals in biomedical devices, in thermal barrier coatings for ceramic matrix composites and C–C composites for hypersonics, and for immobilizing nuclear waste to name a few. For example, boron trioxide, B_2_O_3_, has low glass transition and melting temperatures, a small coefficient of thermal expansion, and is used in many glass formulations (e.g., in borosilicate glasses) to improve hardness and lower water absorption. Also, lead monoxide, PbO, is a common additive to glass that has two stable crystal polymorphs—litharge, which is yellow, is stable at low temperatures, and has a tetragonal structure, and massicot, which is red in color, has an orthorhombic structure, and is stable at higher temperatures (i.e., at and above the transition temperature of 762 K). Metal oxide glasses, like PbO–B_2_O_3_, are often used to immobilize nuclear waste and while PbO does not form a glass by itself, the utility of lead monoxide is that when added to glass formers like boron trioxide, the properties of the resulting metal oxide glass can be tailored by adjusting composition to meet application requirements. For example, the relative compositions of PbO and B_2_O_3_ can be tailored to provide maximum loading of nuclear waste. However, significant structural changes from planar BO_3_ triangles to BO_4_ tetrahedra occur as a function of PbO content above 20 mol% and these structural changes must be considered in order to determine accurate thermophysical properties.

While detailed understanding of the impact of adding metal oxides to glass formers is still an open debate (i.e., whether they function as modifiers or network formers; see^1^, nonetheless they are very useful in tailoring the properties of the glass by varying the composition of metal oxide/glass mixture to meet specific application requirements.

Creating glass formulations experimentally is a time consuming and expensive task. On the other hand, a robust, general-purpose modeling framework for predicting thermomechanical properties of these materials would be valuable in guiding the experimental design of metal oxide/glass formulations. However, glasses like silicon dioxide (SiO_2_), boron trioxide (B_2_O_3_), germanium oxide (GeO_2_) as well as mixture of these glasses with metal oxide additives such as PbO, BaO, Al_2_O_3_, CaO, etc. are among the most difficult amorphous materials to model. There are several reasons for this:

1. Pure glasses and metal oxide glasses frequently exhibit unusual pVT behavior such as maxima in density and coefficient of thermal expansion^2^, negative thermal expansion^3,4^, and minima in Young’s and shear moduli^2^.
2. In certain applications, thermal processing of glass over extended temperature ranges and large temperature gradients is necessary, and this places additional demands on modeling glassy materials (e.g., in hypersonic applications where glassy coatings are used as a thermal barrier to protect SiC and C–C composites. Here temperatures can range from ambient $\to$ 2000 °C and temperature gradients can exist within the aeroshell). These large temperature ranges and gradients can result in changes in glass–ceramic structure and large changes in properties, which present challenges at all modeling length scales.
3. The choice of force fields used in modeling glass and metal oxide glass at the molecular length scale can give poor matches to experimental data because the force field terms do not capture the correct physics and/or the force field parameters are inadequate over the temperature range of interest.

There are very few rigorous computational modeling tools capable of predicting important thermomechanical properties of these materials (e.g., density, glass transition temperature, ${T}_{g}$, softening temperature, ${T}_{s}$, coefficient of thermal expansion, $\alpha$, isothermal compressibility, $\beta$, bulk modulus, $K$, elastic modulus, $E$, enthalpy, $H$, and heat capacity, ${C}_{v}$) with acceptable accuracy and speed. Quantitative values of these properties can play a critical role in material design. For example, matching the coefficients of thermal expansion of substrates and laminates is important in order to avoid delamination on heating. Also, softening temperature is a key design parameter in formulating thermal paints and thermal barrier coatings, which are frequently used in severe, high- temperature environments.

There are several different types of models for glasses and metal oxide glasses: empirical methods, molecular models, and equations of state. While there are several empirical equations of state (EOS) for polymers [e.g., the Hartmann and Hague equation^5^, the double domain Tait (DDT) equation^6^, and the two-domain Schmidt equation^7^, there are far fewer EOS for glasses^8–11^. With the exception of the two-state model of Macedo et al.^10^, most of these EOS are adapted from solid models, relate pressure to volume at constant temperature to quantify shock compression, require many parameters that must be fit to experimental data, and generally provide poor fits to specific volume as a function of temperature. There are also other empirical equations like the method of Gordon and Taylor^12^ and the Fox equation^13^ that can be used to determine glass transition temperature, but these methods do not provide estimates for other thermomechanical properties.

Relevant potential energy (PE) models for glasses and glassy metal oxides generally include electrostatic forces (e.g., Ewald summations) but differ in the way in which dispersion terms are included. Bowron^14^, Bent et al.^15^, Hannon et al.^16^, Hannon et al.^17^, Hannon and Parker^18^, and Hannon et al.^19^ have used the Lennard–Jones 6–12 potential for dispersion terms to model amorphous glass materials such as SiO_2_, GeO_2_, (Cs_2_O)_0.25_(GeO_2_)_0.75_, (Cao)_0.70_(Al_2_O_3_)_0.30_, and (PbO)_0.67_(Ga_2_O_3_)_0.33_. Kuzuu et al.^20^ have used four PE models that include 2- and 3-body terms by combining Morse and Born–Mayer–Huggins (BMH) potentials as well as screened/full electrostatic terms to study anomalous behavior of vitreous SiO_2_. Huang and Kieffer^21^ have developed and used their charge transfer 3-body potential to study thermomechanical properties and densification of vitreous silica and boron trioxide^4^. Soper^22^ developed a method called empirical potential for structure refinement (EPSR) for glassy material and applied it to vitreous boron trioxide^23^. EPSR consists of dispersion forces modeled using the Lennard–Jones 6–12 potential, Coulomb forces computed using Ewald summation, and an additional repulsive exponential potential to prevent atomic overlap. Finally, there is the family of Betrani–Menziani–Pedone^24^ empirical PE models for multicomponent oxide glasses and crystals, which use a Morse potential, a repulsive term, electrostatic forces, a Buckingham repulsive term for network former-network former interactions, and a simple/screened harmonic approximation to 3-body interactions.

While EOS such as the Flory–Orwoll–Vrije (FOV) equation^25,26^, the Sanchez–Lacombe (SL) equation^27,28^, variants of the statistical associating fluid theory (SAFT) of Chapman et al.^29^, the cubic plus association (CPA) equation^30^, and the multiscale Gibbs–Helmholtz Constrained (GHC) equation^31–33^ could, in principle, be used, these rigorous equations of state have not been applied directly to glasses and metal oxide glasses.

Lucia et al.^31^ used the Soave modification of the Redlich–Kwong equation

1 $$p= \frac{RT}{V-b}-\frac{a\left(T,p\right)}{V\left(V+b\right)},$$

and the Gibbs–Helmholtz equation to constrain the energy (or attraction) parameter, $a$, given by

2 $${a}_{L}\left(T,p\right)=\left[\frac{a\left({T}_{c},{p}_{c}\right)}{{T}_{c}}+\frac{{b}_{L}{U}^{DL}}{{T}_{c}ln2}+\frac{2{b}_{L}Rln{T}_{c}}{ln2}\right]T-\frac{{b}_{L}{U}^{DL}}{ln2}-\left[\frac{2{b}_{L}R}{ln2}\right]TlnT,$$

to develop the multi-scale Gibbs–Helmholtz Constrained (GHC) equation of state, where $p$ is pressure, $T$ is temperature in Kelvins, $V$ is the molar volume, $R$ is the universal gas constant, ${U}^{D}$ is the internal energy of departure, $b$ is the molecular co-volume parameter, and ${T}_{c}$ and ${p}_{c}$ are critical temperature and critical pressure, respectively. Given Eqs. (1) and (2) the molar volume and all pVT-related properties (e.g., coefficient of thermal expansion, isothermal compressibility, bulk modulus, etc.) can be computed.

The key attributes of the multi-scale GHC equation are as follows:

1. It uses the Gibbs–Helmholtz equation to constrain the energy parameter.
2. The boundary condition for the energy parameter used to derive Eq. (1) is defined in the conventional way—by setting the first and second derivatives, ($\frac{\partial p}{\partial V}{)}_{Tc}$ and $(\frac{{\partial }^{2}p}{\partial {V}^{2}}{)}_{Tc}$, on the critical isotherm equal to zero. This results in a fixed boundary condition for the energy parameter given by $a\left({T}_{c},{p}_{c}\right)=0.42748\frac{{R}^{2}{T}_{c}^{2.5}}{{p}_{c}}$, which is not applicable to glasses and metal oxide glasses because many of these materials decompose before ever reaching a critical point.
3. Monte Carlo simulations are used to gather only pure component molecular length scale information (i.e., internal energies of departure, ${U}^{D}$) over ranges of temperature and pressure and ${U}^{D}s$ are placed in lookup tables for use in bulk phase modeling and simulation.
4. Monte Carlo simulations need only be run once because ${U}^{D}$ lookup tables can be used over and over again. Thus, the GHC framework is computationally efficient.
5. Mixing rules are used to predict properties of mixtures.
6. The parameters, $a$ and $b$, are never regressed to experimental data. Experimental data is only used to validate predictions by the GHC equation.

Equation (1) has also been used for vapors, hexagonal ice, aqueous electrolytes, and gas hydrates.

The objective of this article is to develop a thermodynamically rigorous modeling framework that addresses the open modeling challenges associated with the design of glassy materials. A general multi-scale equation of state framework for modeling and simulating glassy materials is presented that includes the use of Monte Carlo simulation to gather molecular length scale information and a novel moving boundary equation of state for calculating bulk thermomechanical properties of pure glasses and glassy mixtures. The numerical methodology and simulation results for a pure glass and binary glass mixture are compared to experimental data taken from the open literature where available. This is followed by a discussion of results and the conclusions of this work.

## Materials and methods

### Modeling glasses and their properties with an equation of state

Unfortunately, Eq. (1) breaks down for glassy materials (e.g., asphaltenes, ionic liquids, polymers, pure glass, metal oxide glasses, etc.) because these materials decompose prior to reaching a critical point. This, in turn, makes the boundary condition for the energy parameter in Eq. (1) undefined. To circumvent this Lucia and Gow^32,33^ modified the expression for the energy parameter for pure glasses as follows:

3 $${a}_{G}\left(T,p\right)=\left[\frac{{a}_{0}\left({T}_{g},{p}_{g}\right)}{{T}_{g}}+\frac{{b}_{G}{U}^{DG}}{{T}_{g}ln2}+\frac{2{b}_{G}Rln{T}_{g}}{ln2}\right]T-\frac{{b}_{G}{U}^{DG}}{ln2}-\left[\frac{2{b}_{G}R}{ln2}\right]TlnT,$$

where the quantity ${a}_{0}\left({T}_{g},{p}_{g}\right)$ in Eq. (3) denotes the boundary condition for the energy parameter for pure glassy materials. Lucia and Gow^32^ proposed and used an iterative numerical procedure to estimate ${a}_{0}\left({T}_{g},{p}_{g}\right)$ that minimizes the error between ${U}^{D}$ from Monte Carlo simulation and ${U}^{D}$ calculated from fundamental thermodynamics. However, this numerical procedure is computationally expensive and does not consider ‘soft’ repulsive forces^34^. Therefore, in this work, we propose the following expression for the boundary condition for the energy parameter for glassy materials

4 $$a_{0} \left( T \right) = \left\{ {\begin{array}{*{20}c} { - b_{G} [U^{{DG}} \left( {T_{g} } \right) + [R\left( {\frac{{\ln T}}{{\ln 2}}} \right) + \left( {\frac{{U^{{DG}} \left( T \right)}}{T}} \right)\left] {\left( {T - T_{g} } \right)} \right],~~T \le T_{g} \quad (4{\text{a)}}} \\ { - b_{G} [U^{{DG}} \left( {T_{g} } \right) - [R\left( {\frac{{\ln T}}{{\ln 2}}} \right) + \left( {\frac{{U^{{DG}} \left( T \right)}}{T}} \right)\left] {\left( {T - T_{g} } \right)} \right],~~T > T_{g} \quad (4{\text{b}})} \\ \end{array} } \right.$$

Note that Eq. (4) has the following properties:

1. It is a closed form, temperature-dependent expression for the attraction parameter boundary condition, making it a moving boundary condition.
2. The first term on the right, $-{b}_{G}{U}^{DG}\left({T}_{g}\right)$, is fixed and replaces the computationally expensive numerical procedure described in Lucia and Gow^32^.
3. The second term on the right, ${b}_{G}R\left(\frac{\mathrm{ln}T}{\mathrm{ln}2}\right)(T-{T}_{g})$, is an ideal gas-like contribution that increases the boundary condition as temperature increases.
4. The last term, ${-b}_{G}\left(\frac{{U}^{DG}\left(T\right)}{T}\right)\left(T-{T}_{g}\right),$ is an entropic contribution to attraction due to soft repulsion, where $-(\frac{{U}^{DG}\left(T\right)}{T})\left(T-{T}_{g}\right)={-S}^{D}\Delta T$ and decreases the boundary condition as temperature increases.
5. Regardless of the way in which the boundary condition, ${a}_{0}\left(T\right)$, is computed, the energy parameter, ${a}_{G}\left(T\right)$, satisfies the Gibbs–Helmholtz equation.

Substitution of Eq. (4a) in Eq. (3) gives the following new expression for the energy parameter

5 $${a}_{G}\left(T\right)=\left[\left(\frac{{b}_{G}{U}^{DG}({T}_{g})}{{T}_{g}}\right)\left(\frac{1}{\mathrm{ln}2}-1\right)-\frac{{b}_{G}[R\left(\frac{\mathrm{ln}T}{\mathrm{ln}2}\right)+\left(\frac{{U}^{DG}\left(T\right)}{T}\right)](T-{T}_{g})]}{{T}_{g}}+\frac{2{b}_{G}R\mathrm{ln}{T}_{g}}{\mathrm{ln}2}\right]T-\frac{{b}_{G}{U}^{DG}\left(T\right)}{\mathrm{ln}2}-\left[\frac{2{b}_{G}R}{\mathrm{ln}2}\right]T\mathrm{ln}T.$$

Equation (5) is a closed form and computationally efficient expression for the attraction parameter that accounts for soft repulsive forces through the inclusion of internal energy and entropy of departure terms.

For mixtures, the molecular co-volume parameter, ${b}_{M}$, is computed using

6 $${b}_{M}={\sum }_{i=1}^{C}{x}_{i}{b}_{i}$$

where $M$ denotes the mixture property, $x$ denotes a mole fraction, the subscript $i$ denotes pure component $i$, and $C$ is the number of components in the mixture. Internal energies of departure for mixtures are determined using the linear mixing rule

7 $${U}_{M}^{D}=\sum_{i=1}^{C}{x}_{i}{U}_{i}^{D}.$$

The moving boundary condition for mixtures, ${a}_{0M}$, and the temperature dependent attraction parameter, ${a}_{G}\left(T\right),$ are computed using one fluid theory (i.e., by replacing ${b}_{G}$ with ${b}_{GM}$, ${T}_{g}$ with ${T}_{gM}$, and ${U}^{D}$ with ${U}_{M}^{DG}$ in Eqs. 4 and 5), which gives the following moving boundary condition for ${a}_{0M}$:

8 $$a_{{0M}} \left( {T_{{gM}} } \right) = \left\{ {\begin{array}{*{20}c} { - b_{{GM}} \left[ {U_{M}^{{DG}} \left( {T_{{gM}} } \right) + \left[ {R\left( {\frac{{\ln T}}{{\ln 2}}} \right) + \left( {\frac{{U_{M}^{{DG}} \left( T \right)}}{T}} \right)} \right]\left( {T - T_{{gM}} } \right)} \right],~T \le T_{{gM}} \quad (8{\text{a}})} \\ { - b_{{GM}} \left[ {U_{M}^{{DG}} \left( {T_{{gM}} } \right) - \left[ {R\left( {\frac{{\ln T}}{{\ln 2}}} \right) + \left( {\frac{{U_{M}^{{DG}} \left( T \right)}}{T}} \right)} \right]\left( {T - T_{{gM}} } \right)} \right],~T > T_{{gM}} \quad (8{\text{b}})} \\ \end{array} } \right.$$

and the following expression for ${a}_{GM}\left(T\right)$:

9 $${a}_{GM}\left(T\right)=\left[\left(\frac{{b}_{GM}{U}_{M}^{D}({T}_{gM})}{{T}_{gM}}\right)\left(\frac{1}{\mathrm{ln}2}-1\right)+\frac{{b}_{GM}[-R\left(\frac{\mathrm{ln}T}{\mathrm{ln}2}\right)+\left(\frac{{U}_{M}^{D}\left(T\right)}{T}\right)](T-{T}_{g})]}{{T}_{gM}}+\frac{2{b}_{GM}R\mathrm{ln}{T}_{gM}}{\mathrm{ln}2}\right]T-\frac{{b}_{GM}{U}_{M}^{D}\left(T\right)}{\mathrm{ln}2}-\left[\frac{2{b}_{GM}R}{\mathrm{ln}2}\right]T\mathrm{ln}T.$$

### Molecular length scale information from Monte Carlo simulation

Accurate molecular length scale information is extremely important in building a robust bulk phase model for predicting thermomechanical properties. For the multiscale GHC equation, this means accurate pure component internal energies of departure, ${U}^{D}$, are required. As noted earlier, this information is gathered a priori using Monte Carlo simulations that are run over ranges of temperature and pressure relevant to the application. Results of the Monte Carlo simulations are then placed in pure component lookup tables and used in bulk phase property determinations. In the multiscale GHC framework, pure glass and pure metal oxide properties tend to be very sensitive to values of internal energies of departure. This suggests that large numbers of Monte Carlo cycles and many sets are required to estimate values of ${U}^{D}$.

### Force field models

Force field models play a critical role in computing accurate molecular length scale information and many force fields (potentials) have been used for glasses. Table 1 gives a summary of some of the force field models that have been used to model thermomechanical properties for pure glasses and metal oxide glass mixtures using molecular dynamics and Monte Carlo simulation. As can be seen from Table 1, much of the modeling work in the open literature has focused on silica. However, there is no general agreement on the force field used to model glasses or metal oxide glasses.

**Table 1:** Force fields/potentials that have been used to model various pure glasses and metal oxides.

Glass	Phase	Force field or potential	References
SiO_2_	Liquid	van Best–Kramer–van Santan (BKS)	Shell et al.^3^
	Vitreous	Bohr–Mayer–Huggins (BMH) + Morse potential	Kuuzu et al.^20^
	Vitreous	Charge-transfer three-body potential	Huang and Kieffer^4^
	Vitreous	Lennard–Jones + electrostatic forces	Chu-Cruz et al.^35^
	Vitreous	Lennard–Jones + electrostatic forces	Bowron^14^
	Vitreous	Ab initio-derived Lennard–Jones + electrostatic	Butenuth et al.^36^
	Vitreous	Lennard–Jones + electrostatic forces	Anagnostopoulos et al.^37^
B_2_O_3_	Vitreous	Coordination-dependent charge transfer potential	Huang et al.^21^
	Vitreous	Lennard–Jones + electrostatic forces + repulsion	Soper^23^
	Crystal	Many-body quantum Monte Carlo	Ferlat et al.^38^
GeO_2_	Vitreous	Lennard–Jones + electrostatic forces	Hannon et al.^16^
MO			
PbO	In glass	Lennard–Jones + electrostatic forces	Hannon et al.^17^
	Liquid	Lennard–Jones	Arkundato et al.^39^
BaO	Solid	Lennard–Jones	Xiaoping^40^
Al_2_O_3_	Liquid	Lennard–Jones + electrostatic forces	Soper^41^
	In glass	Lennard–Jones + electrostatic forces	Hannon et al.^19^
CaO	In glass	Lennard–Jones + electrostatic forces	Hannon and Parker^18^

*MO* Metal oxide.

In this work, we have chosen to use Lennard–Jones plus electrostatic forces to model the internal energy of departure

10 $$U^{D} = \mathop \sum \limits_{{i = 1}}^{A} \mathop \sum \limits_{{j = 1}}^{A} \left[ {4\varepsilon _{{ij}} \left[ {\left( {\frac{{\sigma _{{ij}} }}{r}} \right)^{{12}} - \left( {\frac{{\sigma _{{ij}} }}{r}} \right)^{6} } \right] + \frac{{q_{i} q_{j} }}{{4\pi \varepsilon _{0} r}}} \right]$$

where $\varepsilon$ and $\sigma$ are the well depth of the potential function and zero potential distance parameters respectively, $q$ denotes a partial charge, ${\epsilon }_{0}$ is the permittivity, $r$ is the distance between particles (i.e., atoms), $i$ and $j$ are atom indices, and $A$ is the total number of atoms in a molecule.

### Force field parameters

Finding or determining reliable values of the parameters, $\varepsilon$, $\sigma$, and $q$, for glasses and metal oxides can, at times, be quite challenging. Many of the parameters published in the literature [e.g., Si and O parameters in Cruz-Chu et al.^35^, Butenuth et al.^36^, Emani et al.^42^, Anagnostopolous et al.^37^] give values of ${U}^{D}$ and bulk phase properties that vary all over the map.

### Numerical methodology

Before presenting simulation results it is instructive to describe the numerical methodology used in determining thermophysical properties of glass and metal oxide glass, which determines the glass transition and softening temperatures prior to estimating all other desired thermophysical properties. Motivation for the methodology to follow is illustrated using boron trioxide as an example. The zero potential Lennard–Jones parameters, $\sigma$, were taken from Tabrizi et al.^43^ and the well depth parameters, $\varepsilon$, were taken from Soper^23^ and adjusted to remove the EPSR repulsive term. Partial charges were selected to enforce electroneutrality. The B_2_O_3_ force field parameters are shown in Table 2.

**Table 2:** Force field parameters for boron trioxide.

Atom	$$\varepsilon$$ (K)	$$\sigma$$ ($$A)$$	q (eV)
B	47.3029	3.45	+ 0.6
O	50.32	3.47	− 0.4

NVT Monte Carlo simulations with N = 320 atoms at 1 bar pressure were run to gather molecular information. Smaller ensembles will give inaccurate molecular information at higher temperatures.

### Heat capacity

**Figure 1:**
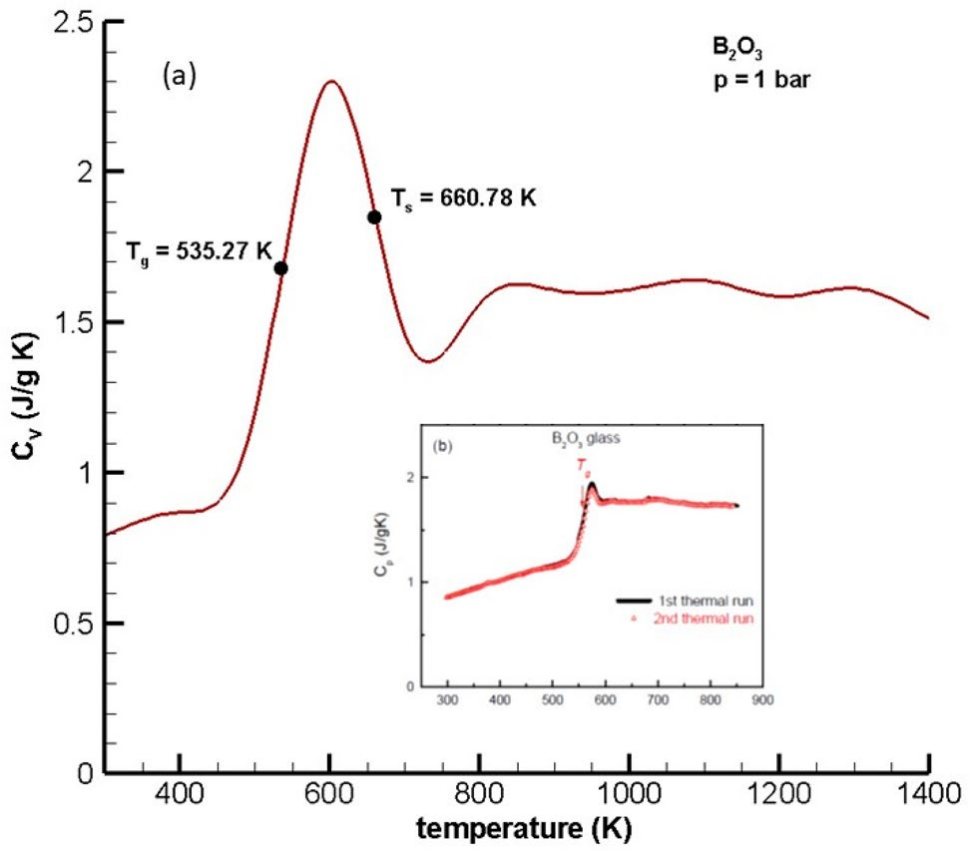
Heat capacity, ${C}_{V}$, vs. temperature for boron trioxide at 1 bar (**a**) Predicted by Monte Carlo simulation. (**b**) Inset: Experimental *C*_*p*_ Data Taken from D’Angelo and Carini^44^.

Figure 1 shows a comparison of the Monte Carlo simulation predictions of specific heat capacity, ${C}_{V}$, vs. temperature for boron trioxide with experimental data from D’Angelo and Carini^44^, where the predicted heat capacity at constant volume was computed using the fundamental expression $(\frac{\partial U}{\partial T}{)}_{V}=\frac{\partial }{\partial T}({U}^{ig}+{U}^{D}{)}_{V}$. For B_2_O_3_ glass, $N$, the number of atoms/molecule, is five and the degrees of freedom, $DoF$, for a polyatomic, nonlinear ideal gas varies from 6 at low temperature to $3N$ at high temperature. Therefore, ${U}^{ig}=(\frac{DoF}{2})RT$ and ${U}^{D}$ comes from the Monte Carlo simulations. However, the results of the Monte Carlo simulations over-predict the maximum heat capacity (i.e., 2.3 J/g K vs. 2 J/g K) and slightly under-predict the heat capacity (i.e., 1.59 vs. 1.75 J/g K) over the range [600–850 K].

### Glass transition and softening temperatures

One of the novel aspects of this work is the conjecture that glass transition and softening temperatures can be estimated using adjacent inflection points on each side of the maximum in ${C}_{V}(T)$. Using the numerical procedure described in the Supplemental Material, adjacent saddle points on each side of the maximum in ${C}_{V}(T)$ give values of ${T}_{g}=535.27 K$ and ${T}_{s}=660.78 K$.

### Volume (or density)

Molar volume is computed directly by solving Eq. (1) with the energy parameter determined by Eq. (5).

### Volumetric coefficient of thermal expansion

Volume versus temperature results from the equation of state computations can be fit and then differentiated to give approximations of $(\frac{\partial V}{\partial T}{)}_{p}$ so that a prediction of $\alpha$ can be computed from the equation

11 $$\alpha =\frac{(\frac{\partial V}{\partial T}{)}_{p}}{V}.$$

Fitting of $V$ vs. $T$ is recommended is because $(\frac{\partial V}{\partial T}{)}_{p}$ involves the attraction parameter derivative, ($\frac{\partial a(T)}{\partial T}{)}_{p}$. This coupled with the fact that derivatives like $(\frac{\partial {U}^{D}}{\partial T}{)}_{p}$ from the Monte Carlo simulation can be inaccurate and sometimes lead to errors in the derivatives $(\frac{\partial p}{\partial T}{)}_{V}$ and $(\frac{\partial p}{\partial V}{)}_{T}$ needed to determine $(\frac{\partial V}{\partial T}{)}_{p}=-\frac{(\frac{\partial p}{\partial T}{)}_{V}}{(\frac{\partial p}{\partial V}{)}_{T}}$.

### Pseudo-algorithm

A general pseudo-algorithm for computing glass transition and softening temperatures along with other thermophysical properties is as follows:

1. Compute all pure component molecular properties (i.e., ${U}_{i}^{D}{^{\prime}}s$, etc.) for the glass under consideration using Monte Carlo simulations over ranges of temperatures and pressures of interest, considering any structural changes that can occur. Typically, four sets of all-atom Monte Carlo simulations with periodic boundary conditions and 100,000 equilibration and 100,000 production cycles are run for each temperature and pressure and averaged. Store the molecular information in pure component lookup tables. Compute the mixture internal energies of departure using Eq. (7).
2. Using internal energies of departure and degrees of freedom compute the total internal energy, $U$, and heat capacity at constant volume, ${C}_{V}$, over the desired range of temperature where $U= {U}^{ig}+{U}^{D}$, ${U}^{ig}=\left(\frac{DoF}{2}\right)RT$, and ${C}_{V}=(\frac{\partial U}{\partial T}{)}_{V}$.
3. Compute the global maximum of ${C}_{V}$ and saddle points on each side of the global maximum using the procedure described in the Supplementary Material. The saddle points on each side of the maximum give predicted values of ${T}_{g}$ and ${T}_{s}$, where ${T}_{s}>{T}_{g}$.
4. Given ${T}_{g}$, compute the boundary condition for the glass using Eq. (4) (or Eq. 8 for mixtures).
5. Using the multiscale GHC equation of state, calculate the molar volume and all desired volume-dependent properties such as volumetric thermal expansion coefficient, isothermal compressibility, bulk modulus, elastic modulus, etc. over the temperature and pressure ranges of interest.

**Figure 2:**
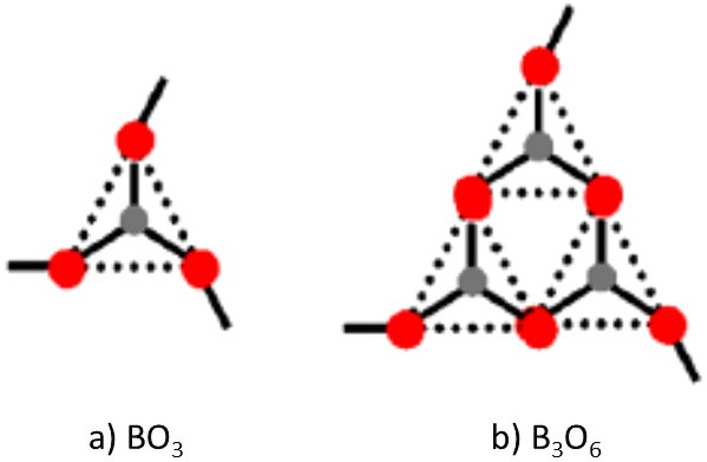
Different structural units of boron trioxide. (**a**) BO_3_ triangles, (**b**) Boroxol rings.

Steps 1 is a critical step of the modeling since it is well known that glasses can exhibit temperature-dependent changes in structure. For example, it is well known that boron trioxide is comprised of two types of elementary building blocks—BO_3_ units and boroxol rings (B_3_O_6_) and that the fraction of boroxol rings decreases with increasing temperature (i.e., from 0.6 at 500 K in the glassy region to 0.2 at 1900 K in the liquid state). See Fig. 2 and Ferlat^38^.

However, it is possible to incorporate structure in the Monte Carlo simulations by using an initial structure in the simulation box that reflects structure. In the case of B_2_O_3_ that would mean initializing the atoms in the simulation box to reflect the ratios of the elementary building blocks BO_3_ to B_3_O_6_.

Step 2 and 3 provide a way of directly calculating glass transition and softening temperatures for pure glasses or metal oxide glasses and set the stage for subsequently computing volume and all volume-dependent thermophysical properties in steps 4 and 5. However, it is important to understand that accurate Monte Carlo simulations must be performed in step 1 to get reliable estimates of ${U}^{D}\left(T\right)$ and the ${C}_{V}(T)$ functionality, particularly at high temperature. This means that several sets of Monte Carlo simulations with a large number of equilibration and production cycles and large ensemble are required.

## Results

To demonstrate the capabilities of the proposed modeling framework, multiscale simulation results for thermomechanical properties of a pure glass and a metal oxide glass mixture are presented and compared to the experimental data in the open literature. All computations were performed on a Dell Vostro 5500 laptop with the Lahey–Fijitsu (LF) 95 compiler using double precision arithmetic.

### Pure glass formers

All-atom boron trioxide NVT Monte Carlo simulations were conducted with N = 320 atoms. Four sets of 100,000 equilibration cycles and 100,000 production cycles each were run for each temperature in the range [200, 1600 K] in 50 K intervals. Results for four sets for each temperature were averaged to provide molecular length scale information.

### Heat capacity

The heat capacity versus temperature curve shown in Fig. 1a shows that the general trend and magnitude of calculated ${C}_{V}(T)$ in this work match the experimental ${C}_{P}(T)$ from D’Angelo and Carini^44^ quite well.However, the results of the Monte Carlo simulations over-predict the maximum heat capacity (i.e., 2.3 J/g K vs. 2 J/g K), slightly under-predict the heat capacity (i.e., 1.59 vs. 1.75 J/g K) over the range [600–850 K] and show a peak slightly to the right of the peak given in the experimental data. Remember, the computed results are truly predictions with no regression to any experimental data. Also, note that there is no clear melting point in Fig. 1a or b because the material is glass. This suggests that the force field model for B_2_O_3_ consisting of Lennard–Jones + electrostatic forces together with Monte Carlo simulation is capable of predicting the heat capacity of boron trioxide with reasonably good accuracy. In addition, it is important to note that the comparison of calculated values of ${C}_{V}$ in Fig. 1a with experimental values of ${C}_{P}$ shown in the Fig. 1b is valid because (a) $\Delta H= \Delta U+ \Delta (pV)$, (b) the $\Delta \left(pV\right)$ term is small, (c) the pressure is low (i.e., 1 bar), and (d) the glass phase is a condensed phase. Thus, $\Delta U\approx \Delta H$ is a good approximation, which implies ${C}_{V}\approx {C}_{P}$.

### Glass transition temperature

The saddle point (inflection point) to the left of the maximum in ${C}_{V}$ shown in Fig. 1a gives a predicted glass transition temperature, *T*_*g*_, of 535.26 K and falls within the range of reported experimental values in the literature [510, 580 K]. More specifically, the experimental value reported by D’Angelo and Carini^44^ is 560 K while other literature values for the glass transition temperature for pure B_2_O_3_ range from 510 K in Ref.^45^ to 571 K in Ref.^46^ to as high as 580 K in Ref.^47^.

### Softening temperature

Softening temperature is generally described as a transport property and characterized as the temperature at which the viscosity, *η*, of the glass is ${10}^{7.6}$ poise (log_10_ (*η*) = 7.6). Experimental viscosity data given in Ref.^48^ in Table II, p. 616 shows that log_10_ (*η*) of B_2_O_3_ has a value of 7.6 at 375 °C (648 K). Like many properties of boron trioxide, there is limited experimental information for softening temperature and in the few articles containing ${T}_{s}$ data^48,53^, the reported results vary widely. Nevertheless, the moving boundary equation of state prediction of softening temperature in Fig. 1a is ${T}_{s}=660.78 \mathrm{K}$, which is in good agreement with the viscosity-based results reported in Table II on p. 616 in Ref.^48^ of 375 °C (648 K)—less than 2% error. However, the estimated softening temperature of B_2_O_3_ reported on p. 379 in Ref.^53^ is much lower—315 °C (588 K) and, in our opinion, is much closer to the glass transition temperature.

### Volume predictions

Table 3 illustrates the volume predictions of the multiscale GHC equation in the glassy region with and without soft repulsive effects.

**Table 3:** Volumes of B_2_O_3_ glass calculated with and without soft repulsion effects in ${a}_{0}\left(T\right)$.

T (K)	Volume^a^ (g/cc)		
	${a}_{0}\left(T\right)=-{b}_{G}{U}^{DG}\left({T}_{g}\right)$	${a}_{0}\left(T\right)=-{b}_{G}{[U}^{DG}\left({T}_{g}\right)+[R\left(\frac{\mathrm{ln}T}{\mathrm{ln}2}\right)+\left(\frac{{U}^{DG}\left(T\right)}{T}\right)](T-{T}_{g})]$	Exp data^b^
301	0.5297	0.5367	0.5474
360	0.5347	0.5411	0.5495
390	0.5376	0.5435	0.5580
505	0.5518	0.5535	0.5598
510	0.5526	0.5540	0.5557
530	0.5557	0.5560	0.5570
	${a}_{0}\left(T\right)=-{b}_{G}{U}^{DG}\left({T}_{g}\right)$	${a}_{0}\left(T\right)=-{b}_{G}{[U}^{DG}\left({T}_{g}\right)-[R\left(\frac{\mathrm{ln}T}{\mathrm{ln}2}\right)+\left(\frac{{U}^{DG}\left(T\right)}{T}\right)](T-{T}_{g})]$	
561	0.5603	0.5621	0.5597
566	0.5611	0.5632	0.5626
581	0.5634	0.5667	0.5657
593	0.5652	0.5697	0.5702
594	0.5654	0.5699	0.5710
623	0.5699	0.5771	0.5777
628	0.5706	0.5783	0.5834
673	0.5777	0.5900	0.5887
723	0.5861	0.5940	0.5984
AAD%^c^	1.53	0.78	

^a^${T}_{g}=535.27$ K; ${b}_{G}=35.752$ cm^3^/mol; ${U}^{D}\left({T}_{g}\right)=$ -1.1975× ${10}^{6}$ cm^6^bar/mol^2^; ${T}_{s}=660.78$ K.

^b^Experimental data from Macedo et al.^49^.

^c^$AAD \%=100\frac{{|V}^{exp}-{V}^{calc}|}{{V}^{exp}}$, where $V$ denotes volume.

**Figure 3:**
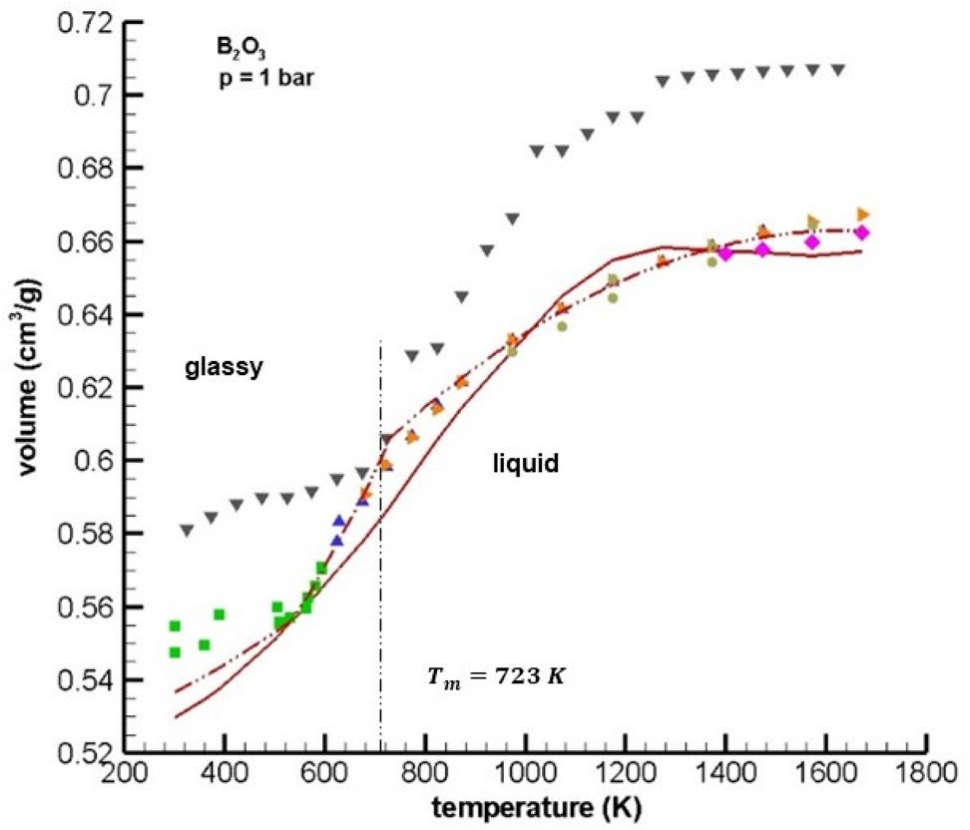
Specific volume vs. temperature for glassy B_2_O_3_ and B_2_O_3_ melt at 1 bar. experimental data: Macedo and Litovitz^10^ (), Napolitano et al.^48^ (), Macedo et al.^49^ (), Roebling^50^ (), Chebykin et al.^51^ (). Simulations: MD Huang and Kieffer^4^ (), multiscale GHC equation without soft repulsion (), moving boundary equation with soft repulsion/temperature-dependent boroxol ring structure ().

Figure 3 shows a comparison of multiscale GHC equation predictions of B_2_O_3_ specific volume with and without soft repulsive forces with experimental volume data for the glassy and melt regions.

The comparison of predicted specific volume with experimental data shown in Table 3 and Fig. 3 are in good agreement with the five experimental data sets from the literature, which contain a total of 51 experimental data points and were taken from^10,48–51^. The values of the AAD % error for the multiscale GHC equation of state without (i.e.,) and with soft repulsion and temperature-dependent boroxol ring structure (i.e.,) were 1.29 and 0.64% respectively for the temperature range [300, 1600 K] and shows that the inclusion of ‘soft’ repulsion and structure in the moving boundary condition and in the Monte Carlo simulations lead to improvements in model predictions of specific volume.Also shown in Fig. 3 are the molecular dynamics (MD) simulation results from^4^ (i.e.,). While the shape of the specific volume versus temperature curve for the MD simulations using the charge-transfer, three-body potential of Huang and Kieffer^4^ shown in Fig. 3 matches the overall shape of the experimental data quite well, the MD simulation predictions over-estimate the specific volume at all temperatures with an AAD% error of 5.92%.

### Volumetric coefficient of thermal expansion predictions

**Figure 4:**
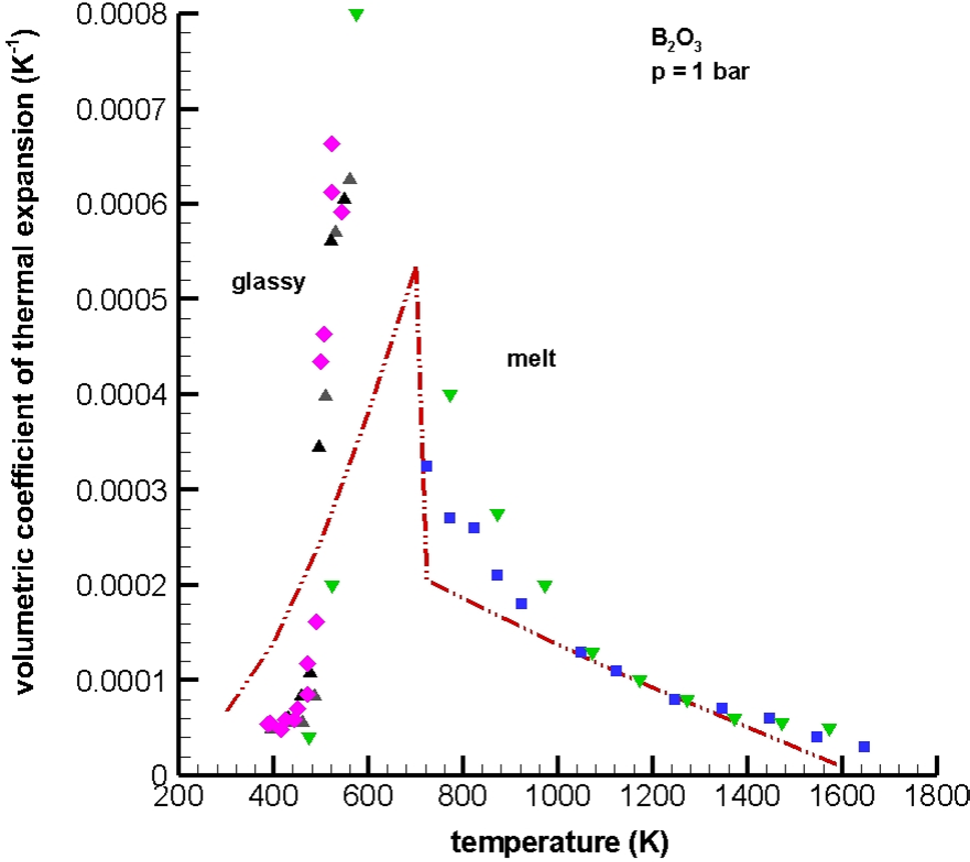
Volumetric coefficient of expansion vs temperature for boron trioxide at 1 bar. Experimental data: Napolitano et al.^48^ (), Spaght and Parks^52^ ()—partially and carefully annealed (sample 1), Spaght and Parks^52^ () —partially and carefully annealed (sample 2), Fajan and Barber^54^ (). Simulation data: moving boundary equation with soft repulsion/temperature-dependent boroxol ring structure ().

Accurate model predictions of volume versus temperature are critical to obtain accurate estimates of the coefficients of thermal expansion. The fits of $V\left(T\right)$ for the moving boundary equation of state shown in Fig. 3 and ($\frac{\partial V}{\partial T})$ used to predict $\alpha$ in this work are given in the Supplementary Material. Figure 4 shows plots of the volumetric coefficient of thermal expansion for the moving boundary equation of state, along with the experimental $\alpha$ data reported in Ref.^52^ and the coefficient of expansion data reported in Ref.^48^, which appears to have been computed from fits of density versus temperature.

There are only a few data sets in the open literature containing experimental coefficient of thermal expansion data for boron trioxide. While there is general agreement with respect to the order of magnitude of $\alpha$ in the range [10^–4^, 10^–5^ °C^−1^], the values of the experimental data vary widely. In Table I, p. 106 Spaght and Parks^52^ provide different sets of results for $\alpha$ for two separate samples of B_2_O_3_ glass for partially and carefully annealed samples in which volume was measured using dilatometry and $\alpha$ was approximated using the finite difference expression $\mathrm{\alpha }=(\frac{2}{{V}_{1}+{V}_{2}})(\frac{\Delta {V}_{B2O3}}{{T}_{2}-{T}_{1}})$. However, Spaght and Parks present no volume data or experimental error statistics for the coefficients of expansion—so it is difficult to judge the accuracy of their data. However, for partially annealed and carefully annealed boron trioxide, Spaght and Parks show that $\alpha$ increases with increasing temperature over the range [122, 275 °C] corresponding to temperatures up to the glass transition temperature. Again, it is important to note that these authors present no raw volume data in their paper. Donoghue and Hubbard^53^ used interferometry and report a melting point of 450.8 °C (see p. 375) and a softening temperature of 315 °C (see p. 379). However, they only describe the qualitative behavior of $\alpha$ for B_2_O_3_. Fajans and Barber^54^ use electronic structure arguments and the experimental data in Ref.^53^ and in Ref.^55^ respectively to sketch plots of $\alpha (T)$ similar to those given in Ref.^52^ for glassy B_2_O_3_ and in Ref.^48^ for liquid B_2_O_3_. Finally, in Fig. 3, p. 616 in Ref.^48^ it is shown that $\alpha$ for boron trioxide decreases monotonically with increasing temperature over the range [400, 1400 °C], which corresponds to temperatures in the melt regime.

The moving boundary equation of state predictions of volumetric coefficient of thermal expansion are in good agreement with experimental $\alpha$ data reported in Refs.^48,52,54^ both in temperature trends and in order of magnitude [10^–4^, 10^–5^ °C^−1^]. Like the experimental data, for which there is considerable scatter, the moving boundary equation predictions show a rapid rise in $\alpha \left(T\right)$ with increasing temperature in the glassy region and a decline in $\alpha \left(T\right)$ with increasing temperature in the melt region.

### Metal oxide glass: lead monoxide-boron trioxide

Mixtures of lead monoxide and boron trioxide have been studied by Refs.^56–61^. To begin, to calculate glass transition and softening temperatures for mixtures of PbO and B_2_O_3_ and generate ${C}_{VM}(x,T)$ vs. temperature curves similar to Fig. 1a, a force field and force field parameters for PbO are needed. Table 4 gives the Lennard–Jones 6–12 parameters and partial charges for PbO, which were taken from Refs.^62,63^. We first tested the PbO force field parameters by determining internal energies of departure as a function of temperature. We then used those internal energies of departure to calculate the densities of litharge and massicot using the moving boundary equation of state. In each case the calculated predictions were close to values reported in the literature. More specifically, literature values of densities for litharge and massicot range from 9.14–9.53 and 9.56–10 g/cm^3^ respectively (e.g., mindat.org) while the moving boundary equation of state predictions were 9.13 g/cm^3^ at 300 K for litharge and 9.58 g/cm^3^ at 800 K for massicot.

**Table 4:** Force field parameters for lead monoxide.

Atom	$$\varepsilon$$(K)	$$\sigma$$ ($$A)$$	q (eV)
Pb	430.29814	4.404	+ 1.218
O	39.4556	3.627	− 1.218

### Heat capacity vs. temperature

**Figure 5:**
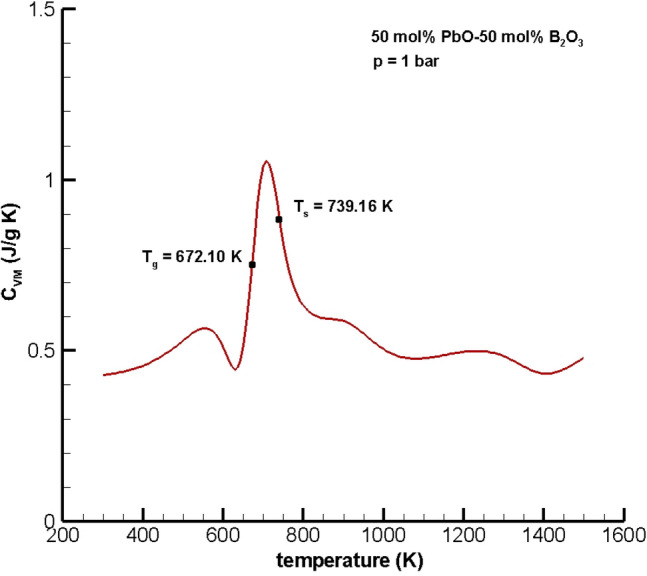
Heat capacity, ${C}_{V}$, vs. temperature for 50 mol% lead monoxide-50 mol% boron trioxide glass at 1 bar predicted by Monte Carlo Simulation using the linear mixing rule for ${U}_{M}^{D}$.

Figure 5 shows a plot of ${C}_{VM}(x,T)$ vs. temperature at 1 bar for a mixture of 50 mol% PbO and 50 mol% B_2_O_3_ over the temperature range [300, 1500 K], where ${C}_{VM}(x,T)$ was calculated using the finite difference formula

13 $${C}_{VM}\left(x,T\right)=\frac{{U}_{M}\left(x,T+\Delta T\right)-{U}_{M}\left(x,T\right)}{\Delta T},$$

where ${U}_{M}(x,T)= {U}_{M}^{ig}(x,T)+{U}_{M}^{D}(x,T)$, ${U}_{M}^{ig}\left(x,T\right)=\sum_{i=1}^{2}{x}_{i}(\frac{{DoF}_{i}}{2})RT,$ and ${U}_{M}^{D}(x,T)$ was calculated using NVT Monte Carlo simulations with 25,000 equilibration cycles and 75,000 production cycles. Also, the degrees of freedom, $DoF$, for the ideal gas contribution to the total internal energy were ${DoF}_{PbO}=9$ because PbO is a triatomic linear molecule and ${DoF}_{B2O3}=15$ since B_2_O_3_ is a polyatomic nonlinear molecule.

The shape of ${C}_{VM}(x,T)$ vs. temperature for the equimolar mixture of PbO and B_2_O_3_ is qualitatively the same as that for pure boron trioxide but is shifted to higher temperatures due to the presence of the network former/modifier PbO. Also, the peak in the heat capacity of the mixture is less than that of pure B_2_O_3_ by about half because the heat capacity of lead monoxide is small (0.2 J/g K) compared to that of pure boron trioxide (2 J/g K).

### Glass transition and softening temperatures of PbO–B_2_O_3_

Glass transition and softening temperatures for mixtures are a function of composition and temperature. For mixtures of PbO and B_2_O_3_, the addition of the network former/modifier PbO increases the glass transition and softening temperatures. Calculation of the inflection point to the left of the maximum in ${C}_{VM}(x,T)$ at $T=706.40 K$ using the procedure described in the Supplemental Material gives an estimate of the glass transition temperature of ${T}_{g}=672.10 K$.Calculation of the inflection point to the right of the maximum at $T=706.40 K$ yields a softening temperature of ${T}_{s}=739.16 K$.

### Volume and volumetric coefficient of thermal expansion

While there is some linear thermal expansion coefficient data reported in Table 3, p. 591 in Ref.^66^, there is no volume or density data. There is also limited experimental values of density and linear thermal expansion coefficients measured by gamma densitometry in Table 2, p. 11 in Ref.^67^ for three different molar compositions of PbO and B_2_O_3_. Moreover, the data in Ref.^66^ and in Ref.^67^ are easily reconciled and provide consistent values of linear thermal expansion coefficients. Here the focus for comparisons of modeling results with experimental data will be an equimolar mixture of PbO and B_2_O_3_.

From the calculated glass transition temperature, ${T}_{g}=672.10 K$, and the linear mixing rules for molecular co-volume and internal energy of departure given by Eqs. (6) and (7) respectively, molar volumes and volumetric coefficients of thermal expansion for 50–50 mol% (76.2–23.8 wt%) PbO–B_2_O_3_ were calculated using the moving boundary equation of state. The predictions and experimental data are shown in Table 5, in which the volumetric coefficients of thermal expansion were computed analytically from 25 to 300 °C and averaged.

**Table 5:** Comparisons of model predictions with experimental data for 50–50 mol% PbO–B_2_O_3_.

Thermophysical property	Geller and Bunting^66^	Drotning^67^	Model predictions
Density @ 800 °C (g/cm^3^)		5.03^a^	5.27
$\alpha$ (K^−1^)	2.70 × 10^–5b^	2.85 × 10^–5a,c^	3.79 × 10^–5^

^a^Polynomial extrapolation of experimental data.

^b^Interpolated average from room temperature to 250 °C.

^c^Mean value from 50 to 300 °C.

The results in Table 5 show that the moving boundary equation of state gives good estimates of specific volume and average volumetric coefficients of expansion for a 50–50 mol% mixture of PbO and B_2_O_3_. The error in the volume prediction at 800 °C is 4.77% while the average value of the volumetric coefficient of expansion, while higher than the experimental values, is certainly the right order of magnitude.

### Glass transition temperature

The calculated glass transition temperature for a 50–50 mol% mixture of PbO and B_2_O_3_ from Fig. 5 is within 3.67% of the experimental glass transition temperatures of 659 K reported in Table 1, p. 369 in Ref.^64^ and 658 K reported in Fig. 1, p. 749 in Ref.^65^. In contrast, a glass transition temperature of ${T}_{g}=678 K$ for an equimolar mixture of PbO and B_2_O_3_ is reported in Ref.^58^.

### Softening temperature

Experimental data for softening temperature for an equimolar mixture of PbO and B_2_O_3_ available in the open literature is in Table 3, p. 591 in Ref.^65^ where the authors report ‘beginning of softening’ temperatures for a range of composition of PbO–B_2_O_3_—but not specifically for a 50–50 molar mixture. Interpolation of the values in Ref.^65^ for an equimolar mixture (76.22 wt% PbO) yields an estimated experimental softening temperature of 685.85 K (412.7 °C). In Fig. 1 in Schwarz and Ticha^68^ the softening temperature for a molar ratio of PbO/B_2_O_3_ equal to 1 is 718 K (450 °C) so there is some disagreement in the experimental data in references^65,68^. The softening temperature predicted by the moving boundary equation of state is 739.16 K is quite a bit higher than the experimental value. Nonetheless, the error in the modeling prediction is 12.92% when compared to the data in Ref.^66^, which is reported as the ‘beginning of softening’, and 3.55% when compared to the data in Ref.^68^. Moreover, it is unclear what Geller and Bunting^65^ mean by ‘beginning of softening’.

## Discussion and conclusions

A moving boundary condition for the multiscale Gibbs–Helmholtz equation of state that accounts for soft repulsion was proposed. This new boundary condition gave rise to a moving boundary equation of state, which was used to determine thermophysical properties of pure boron trioxide and an equimolar mixture of PbO–B_2_O_3_. The predicted glass transition temperature for B_2_O_3_, 535.27 K, was within the range of experimental values of [510, 580 K] reported in the literature, while the predicted softening temperature of 660.78 K was within 1.97% of the experimental value reported in Napolitano et al.^48^. The moving boundary equation of state prediction of glass transition temperature for a mixture of 50 mol% PbO and 50 mol% B_2_O_3_ was 672.10 K, which is within the range reported in the literature [658, 678 K]. For pure boron trioxide, the equation of state predictions of volume was within 0.64% and volumetric coefficients of thermal expansion were in the same range as those reported in the literature. Equation of state predictions of the same thermophysical properties for an equimolar mixture of lead monoxide and boron trioxide were also good but comparisons were limited to very few available experimental data.

It is also important for the reader to understand that there is limited experimental data for metal oxide glasses in the open literature and, in many cases, there is considerable disagreement among the experimental data. Moreover, because none of the papers used for comparison in our manuscript presents error bars for the experimental data or gives any statistical information regarding experimental errors and with such wide variation in the experimental data for various properties, it is difficult to identify the source of the disagreement between experiments and modeling. In our opinion, what is important from a modeling and simulation perspective is for the modeling framework to give results that (1) fall within the range of published experimental data (i.e., have the right order of magnitude) and (2) show the correct temperature trends. However, this is not to imply that there are not errors introduced in modeling. While computations should not be expected to match experimental data for different properties to the same level of accuracy, the proposed computational framework can introduce errors due to errors in the (1) Monte Carlo simulations of internal energies of departure, and (2) the mixing rules for mixture internal energies of departure and molecular co-volume.

In conclusion, it was shown that glass transition and softening temperatures can be estimated by computing adjacent saddle points on ${C}_{V}\left(T\right)$ vs. temperature, where ${C}_{V}\left(T\right)$ is computed from finite difference derivatives of the total internal energy. Internal energies of departure from Monte Carlo simulations needed to compute ${C}_{V}\left(T\right)$ were used within the multiscale GHC equation of state framework and provided good estimates of volume and volumetric coefficients of thermal expansion.

Finally, the modeling and simulation framework presented in this work can be readily extended to other metal oxide glasses and has the potential to guide the experimental design of these materials.

## Supplementary Information

Supplementary Information.

## Data Availability

Input and output data from all numerical simulations and plots presented in this paper are available by contacting the corresponding author.
