# Supplementary material for: Modeling thermophysical properties of glasses
Source: Sci Rep. 2023 Jan 18;13:989. doi: 10.1038/s41598-023-27747-5 (PMC9849270; doi:10.1038/s41598-023-27747-5)
Supplement: Supplementary file 1 — Supplementary Information. [file 41598_2023_27747_MOESM1_ESM.docx]

**Appendix A: Numerical Procedure for Computing Minima of** $\boldsymbol{C}_{\boldsymbol{v}}\boldsymbol{(T)}$

For any given composition, $x$, and pressure, $p$, the numerical procedure for determining minima on $C_{v}(T)$ is as follows:

1. Choose temperature, $T$, and temperature increments, $\Delta T$.
2. Use the multi-scale GHC equation to determine volume, $V(T)$ and total potential energy, $U\left( T \right)=(U^{ig}+U^{D})$, and $C_{v}(T)$.
3. Set $T=T+\Delta T$and go to step 2).
4. Repeat steps 2 through 4 for the desired temperature range.
5. Plot $C_{v}(T)$ vs. $T$ and fit to a cubic spline over each temperature interval using a sufficient number of knots.
6. From step 5) $C_{v}(T)$ on each temperature interval, $\Delta T$, is given by the following cubic spline polynomial

$C_{v}\left( T \right)=a+bT+cT^{2}+dT^{3}$ (A1)

1. Calculate all stationary points of $C_{v}\left( T \right)$ in Eq. A1 on the interval $\Delta T$ using the following formulae

$\frac{d(C_{v}\left( T \right))}{dT}=b+2cT+3dT^{2}=0$ (A2)

1. The solutions to Eqs. A2 and A3 are the following

$T_{1},T_{2}= \frac{-c\mp\sqrt{c^{2}-4bd}}{2b}$ (A3)

The maxima on $\Delta T$ correspond to the ${T^{*}=T}_{1}$ or ${T^{*}=T}_{2}$ for which the second derivative

$\frac{d^{2}(C_{v}\left( T \right))}{dT^{2}}=2c+6dT<0$ (A4)

1. Repeat steps 6) through 8) for all temperature intervals.

**Appendix B: Fits of Computed Volume versus Temperature**

In this appendix, curve fits of boron trioxide volume predictions versus temperature using the moving boundary equation of state are given. Derivatives of volume with respect to temperature at constant pressure are obtained from differentiation of the volume expressions for two separate temperature regimes – for $T\leq T_{m}$ and $T>T_{m}-$ corresponding to the glassy and melt regimes, respectively.

Glassy Regime

For $T\leq T_{m}$

${V=0.5309+3.658x10}^{-5}T-1.537x{10}^{-7}T^{2}+3.387x{10}^{-10}T^{3}$ (B1)

$(\frac{\partial V}{\partial T})_{p}=3.658x{10}^{-5}-3.074x{10}^{-7}T+1.0161x{10}^{-9}T^{2}$ (B2)

Melt Regime

For $T>T_{m}$

$V=0.4798892375573+2.227999057612x{10}^{-4}T-6.770589163281x{10}^{-8}T^{2}$, (B3)

($\frac{\partial V}{\partial T})_{p}=2.22799905x{10}^{-4}-1.35411783x{10}^{-7}T$ (B4)
